# Recognizing the cultural background, motivation, and experience of TN-visa workers in the U.S. swine industry

**DOI:** 10.1093/tas/txag047

**Published:** 2026-04-22

**Authors:** Magdiel Lopez-Soriano, Talita P Resende, Andréia G Arruda, Magnus R Campler, Isaiah Franco, Kara Flaherty, Anna K Johnson, Monique Pairis-Garcia, Samira Chatila, Maria Pieters, Pedro Urriola, Kelly L Adams, Nahida Begum, Douglas Jackson-Smith, Timothy J Safranski

**Affiliations:** Extension Department- Swine, University of Missouri, Columbia, MO, 65211, United States; College of Food, Agriculture, and Environmental Sciences, The Ohio State University, Wooster, OH, 44691, United States; College of Veterinary Medicine, The Ohio State University, Columbus, OH, 43210, United States; College of Veterinary Medicine, The Ohio State University, Columbus, OH, 43210, United States; School of Environment and Natural Resources, The Ohio State University, Wooster, OH, 44691, United States; College of Veterinary Medicine, The Ohio State University, Columbus, OH, 43210, United States; Department of Animal Science, Iowa State University, Ames, IA, 50011, United States; Department of Population Health and Pathobiology, NC State University, Raleigh, NC, 27606, United States; Department of Veterinary Population Medicine, University of Minnesota, St. Paul, MN, 55108, United States; Department of Veterinary Population Medicine, University of Minnesota, St. Paul, MN, 55108, United States; Department of Veterinary Population Medicine, University of Minnesota, St. Paul, MN, 55108, United States; Assessment Resources Center, College of Education & Human Development, University of Missouri, Columbia, MO, 65211, United States; Assessment Resources Center, College of Education & Human Development, University of Missouri, Columbia, MO, 65211, United States; School of Environment and Natural Resources, The Ohio State University, Wooster, OH, 44691, United States; Extension Department- Swine, University of Missouri, Columbia, MO, 65211, United States

**Keywords:** emigration and immigration, foreign professional personnel, Hispanic or Latino, labor force, personnel turnover, personnel management

## Abstract

The U.S. swine industry faces ongoing challenges in hiring and retaining a skilled workforce. Therefore, it heavily relies on foreign workers from the North American Free Trade Agreement (NAFTA) through the TN-visa program. TN-visa workers also experience high turnover rates, which shows a strong need to examine the retention of these workers in greater detail. The objectives of this research were to determine the demographics, motivation, and experience of this critical workforce. Furthermore, it was to determine predictive variables of career advancement and role attainment. A survey was conducted with 261 TN-visa workers. Thirty farms surveyed reported approximately 40% of TN-visa employees within their workforce. In addition, two-thirds of TN-visa holders move to the U.S. in their early to mid-thirties, have high educational attainment (10% earned a master’s degree) and prefer to speak Spanish (80%). They expressed the desire for better salaries, and financially supporting family in Mexico was the primary driver for employment in the U.S. Professional motivations were highly rated as acquiring new skills and experiencing a positive and supportive work environment. Cultural adaptation was the greatest obstacle for resettling workers, particularly navigating language barriers, which hindered their ability to communicate effectively in the workplace and integrate into the local community.

The multivariable mixed-effects logistic regression revealed that TN-visa workers with 3–6 years and over 6 years of experience in the TN-visa program demonstrated three-fold (OR = 3.15; *P* = 0.005) and four-fold (OR = 4.00; *P* = 0.014) elevated odds of holding managerial positions, respectively, in comparison to those with less experience in the program. Moreover, employees that were aware of promotion opportunities at their workplace had over four-fold higher odds of working in upper management compared to workers that did not (OR = 4.4; *P* = 0.003). Also, females tended to have two-fold higher odds of holding a management position compared to males (OR = 2.00; *P* = 0.065). Furthermore, extensive organizational support designed to mitigate the social and linguistic obstacles associated with cross-border professional relocation is crucial for enabling TN-visa professionals to effectively integrate and thrive in their positions. Addressing these systemic issues will be crucial for the U.S. swine industry to stabilize its workforce and fully leverage the expertise of these skilled employees.

## Teaser

This study provides new insight into the backgrounds, motivations, and workplace experiences of TN-visa workers in the U.S. swine industry, showing that retention depends not only on wages, but also on language accessibility, work environment, and visible opportunities for advancement.

## Graphical Abstract

**Graphical Abstract:**
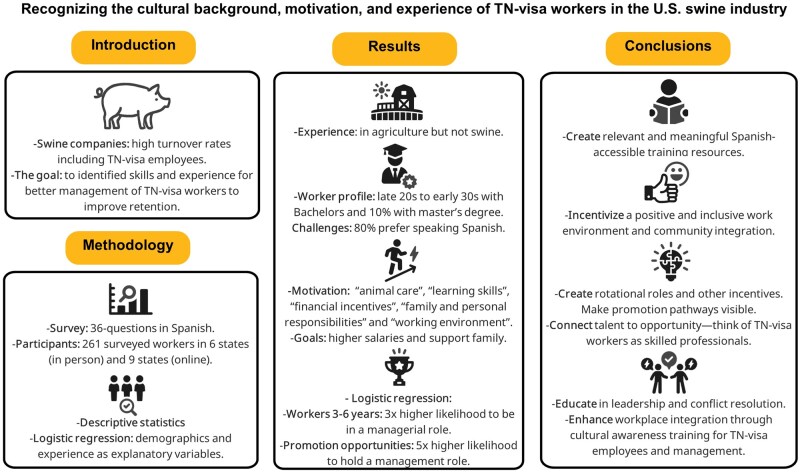

## Introduction

The nonimmigrant North American Free Trade Agreement (NAFTA) visa program, previously referred to as the Trade NAFTA Professional (TN) visa, has become an increasingly important mechanism through which U.S. swine operations recruit qualified Mexican professionals in response to persistent labor shortages. Signed in 1994, this program has provided a simplified route for qualified experts from Canada and Mexico seeking employment in the United States in a range of eligible disciplines on a TN-visa (Shih 2024). TN visas allow professionals to work in the U.S. in renewable three-year increments, providing swine operations with a stable source of skilled labor for prearranged positions. The TN visa program provides employers with access to year-round skilled labor aligned with the operational demands of swine production.

Broader structural shifts in the rural U.S. labor market must inform our understanding of the expansion of TN-visa employment in swine production. Swine producers have faced increasing difficulty recruiting and retaining domestic labor, shaped by persistently low unemployment, rural outmigration, demographic aging, and changing educational and occupational trajectories in nonmetropolitan communities (Johnson 2022; Miller 2023; U.S. Department of Commerce 2023; Palange 2025; U.S. Department of Agriculture—Economic Research Service 2025). Within this context, the TN visa has become one mechanism through which employers secure a highly skilled workforce in a production system that depends on labor with broad knowledge and expertise.

Specific to the swine industry, the TN visa program has been instrumental in facilitating the hiring of Mexican professionals classified under occupations such as “animal breeders” (Yarian et al. 2022; Ramos and Reynaga 2023). However, the availability of TN workers may be evolving in response to broader policy and political dynamics. While TN visa issuances have generally increased over time—with the exception of a temporary decline in 2020 (Shih 2024)—recent data indicate a notable contraction. In 2024, approximately 16,000 TN visas were issued across all eligible occupations, representing a 53% decrease compared to 2023 (U.S. Department of State 2025). Although these fluctuations may have implications for labor availability, the underlying drivers of visa issuance trends fall outside the scope of this study.

It important to highlight that TN-visa workers in swine production also occupy a distinct position relative to other immigrant labor populations in U.S. agriculture. Unlike many agricultural workers employed through other labor pathways, TN-visa professionals must meet formal educational requirements tied to visa eligibility, which places them in a markedly different credentialed category. At the same time, formal qualification does not necessarily translate into seamless workplace incorporation. English proficiency is not a prerequisite for TN visa approval, and many workers may enter employment more comfortable communicating in Spanish than in English. As a result, TN-visa workers may simultaneously be highly educated and professionally qualified while still navigating linguistic and cultural barriers that shape training access, communication with supervisors, and the everyday experience of work.

Despite the growing importance of TN-visa workers in U.S. swine production, these professionals remain largely absent from the research-based literature on agricultural labor, retention, and workforce development. Much of the existing discussion treats TN workers primarily as a response to labor shortages or as a legal mechanism for hiring reliable labor. One critical gap concerns their retention: evidence suggests comparatively high turnover and mobility across livestock industries, patterns that may distinguish TN-visa workers from other agricultural labor groups. More broadly, U.S. swine operations continue to face persistent challenges in retaining employees despite ongoing incentive and management efforts. Indirect evidence indicates that TN-visa workers are not exempt from these dynamics (National Pork Board 2022), yet the fundamental drivers remain inadequately examined. Given their demonstrated role in sustaining the U.S. livestock sector, it is essential to identify the factors shaping retention among TN-visa workers, assess their relative importance, and examine how elements such as workplace integration and sense of belonging influence their long-term employment outcomes.

The present study addresses these gaps by examining TN-visa workers in U.S. swine production as an understudied workforce population whose retention may depend not only on wages but also on organizational conditions, including language accessibility, work environment, professional development, and opportunities for advancement. Using survey data collected from TN-visa workers across major pork-producing states, the study pursues three objectives. First, it documents workers’ demographic, educational, cultural, and occupational characteristics. Second, it examines their motivations for employment in the U.S. swine industry and the barriers they face in resettlement and long-term retention. Third, it analyzes factors associated with career progression, particularly the attainment of managerial roles.

By addressing these objectives, the study aims to contribute a more analytically grounded understanding of TN visa workers not simply as labor inputs but as skilled migrant professionals whose integration and retention are increasingly consequential to the sustainability of U.S. swine production systems.

## Materials and methods

### Institutional oversight

All research was reviewed and approved by the University of Missouri’s Institutional Review Board Committee for Human Subjects Research (2101146-MU) and deemed exempt by the Ohio State University Institutional Review Board (2024E0680).

### Survey design

The Assessment Resources Center (ARC) at the University of Missouri facilitated the design of the 36-question survey. This study utilized a descriptive survey with a predominantly quantitative design, consisting of 30 structured fixed-choice items. Six open-ended questions organized across seven core themes: (1) general farm information, (2) demographics, (3) cultural, professional, and academic backgrounds (4) personal and professional goals, (5) training opportunities, (6) improvements to work conditions and (7) factors that drive retention. The survey was designed based on on-farm surveys (Campler et al. 2020; Black and Arruda 2021; Yarian et al. 2022) and was reviewed by all co-authors for appropriate language to ensure the survey adequately captured these themes.

The survey included closed- and open-ended questions, 5-point Likert-type rating items, multiple-choice items (including check-all-that-apply formats), numeric entry questions, and skip-logic items. Rating questions were formulated in following categories: (1) reasons to come to the U.S. under a TN-visa, (2) professional aspirations, and (3) personal goals, several themes were provided in each category. Participants were asked to rate each item using a 5-point Likert-type scale ranging from “not important” to “very important.” For resettlement barriers, respondents indicated how difficult they found each challenge using a 4-point scale ranging from “not difficult” to “very difficult.”

The survey was translated into Spanish by a native Spanish-speaking member of the research team (MLS). Prior to survey dissemination, the research team solicited feedback from several bilingual human resources professionals and swine technical trainers from private swine companies for accuracy and comprehensibility. The survey was designed to be completed in less than 35 min. Once the survey was reviewed, it was initially implemented at small group meetings held on or near participating farms. The Principal Investigator (PI) of the project (MLS) led these sessions. A copy of the survey instrument was included in supplementary material, available as *Translational Animal Science* online.

### Sampling

A formal sample size was not calculated, as we attempted to capture the largest sample size possible across as many different U.S. states as possible. To guide recruitment, the research team initially set a target of approximately 30 surveys per state. Convenience sampling was chosen due to the unpredictability over participants’ accessibility, availability, or willingness to get involved, rather than through random or systematic selection.

### In-person data collection

Fieldwork was conducted in person between July 2024 and April 2025. The survey was initially conducted in-person with TN-visa-holding workers in six top pork-producing states: Iowa, Illinois, Minnesota, Missouri, North Carolina, and Ohio. Collaborating researchers contacted human resource offices, owners, or farm managers of large swine systems, contract growers, and independent producers to identify TN-visa workers who were eligible to participate.

After TN-visa workers were identified, they were invited to attend an in-person meeting. Most of the in-person sessions were held at a neutral location, such as a local restaurant or a county library, with no supervisor present while the survey was being completed. All employees were told about the promised anonymity, and, depending on the location, light refreshments were provided.

Upon arrival, TN visa workers were familiarized with the study, the consent form was reviewed, the utilization of their information was explained, and thereafter they were encouraged to take a paper survey in either Spanish or English. During most of the sessions, a team member (MLS) read each question; however, in certain instances, participants were permitted to complete the survey independently, with a team member ready to address any inquiries (IF).

Participants were allotted around 1–3 min to answer each question after they were consulted to determine if additional time was needed. Sessions lasted for around 45 min. Once participants finished, a $25 gift card was handed out along with light refreshments in appreciation for their time and willingness to contribute.

During data collection, farm managers, production managers, and human resource professionals were asked by the research team to estimate both the current number of TN-visa workers employed by the farm and the total number of hired workers. This information was collected to determine the proportion of the farm labor force that is employed under a TN visa and thereby estimate TN visa usage in the swine industry.

### Online survey

After the fieldwork was conducted, the online survey was uploaded utilizing Qualtrics (Provo, Utah, U.S.) to enable additional responses. The team opted not to establish a target number of online responses to be obtained but instead aimed to broaden geographic coverage and improve descriptive breadth of the sample. The QR codes were distributed via human resources managers, farm managers, and Pork State Associations across the six targeted states, who were invited to share a link to the survey with their TN-visa workers. In addition, the survey was posted on LinkedIn (Sunnyvale, California, U.S.) and several TN-visa groups in Facebook (Menlo Park, California, U.S.). The online version remained open for 30 consecutive days. No monetary incentives were offered to online participants due to anonymity requirements. All data collected was kept by the ARC to ensure a prominent, appropriate level of security and anonymity for the respondents.

### Data entry and analysis

All data obtained from the in-person survey were translated into English and manually transcribed into Microsoft Excel (Redmond, Washington, USA) by the PI’s research group.

To ensure data quality, all transcriptions underwent a two-step review: a team member (AGR) verified alignment with participant responses, and a statistician (MRC) assessed completeness and consistency with the original database. Discrepancies were resolved through discussion, with final verification by the project’s PI when needed; survey data exported from Qualtrics underwent the same quality assurance procedures. Data from the online survey was directly exported from the Qualtrics platform and compiled into an MS Excel spreadsheet. The same process of data quality assurance for the in-person surveys was performed for the online questionnaire.

None of the in-person surveys were excluded, but approximately 21% of the online surveys were eliminated from the statistical analysis because of incompleteness, indicating that fewer than 80% of the total questions were answered. Data was analyzed across key demographic variables such as age, education, and length in the TN visa program. Percentages and frequency counts were used to represent categorical data (e.g., sex, position held, degree earned). Open-ended responses (motivation, top three aspects to work on a farm, professional aspirations, professional goals, and resettling barriers) were qualitatively coded into qualitative thematic categories to complement quantitative findings.

### Qualitative coding for employment motivation in swine production

Participants were asked two open-ended questions to assess work-related motivations and perceptions. First, respondents identified the top three aspects they valued most about working at a swine farm. Second, they were asked, “Why do you come to work at the farm every day?”

Responses were analyzed using thematic content analysis. An initial coding framework was developed inductively based on recurring patterns in the data. Three co-authors (AGA, MC, DJS, and IF) independently reviewed a subset of responses to identify preliminary themes, which were then discussed and refined into a shared coding scheme. The full dataset was subsequently coded using this framework. Discrepancies in coding were resolved through discussion until consensus was reached.

Primary themes for motivations related to working at a swine farm included “animal care work,” “work environment & conditions,” and “learning &career development.” Key drivers of daily attendance included “financial incentives,” “learning &career development,” and “family & personal responsibilities.”

### Logistic regression analysis

Associations between worker characteristics and managerial role attainment were evaluated using logistic regression. The binary outcome variable indicated whether a respondent held an upper management position (yes/no) or an hourly role.

Prior to the logistic regression model development, Spearman rank correlation coefficients were calculated to assess multicollinearity among explanatory variables, using a threshold of ≥ 0.6. Variables considered included years in the TN-visa program (<3, 3–6, > 6 years), age group (18–34, 35–44, 45–65), previous swine experience (yes/no), prior agricultural experience other than swine (categorized as cattle/equine, poultry, small ruminants, or crops/greenhouse), sex, educational background, promotion opportunity awareness (yes/no), and willingness to apply for promotion (yes/no). Univariate logistic regression models were first fitted for each explanatory variable. Variables with *P* < 0.20 were considered for inclusion in the multivariable model.

A multivariable mixed-effects logistic regression model was then constructed to identify factors associated with holding a managerial position. Respondents’ home state was included as a random effect. Variable selection was guided by backward stepwise elimination, with confounding assessed based on a ≥ 20% change in coefficient estimates. Model fit was evaluated using Akaike’s Information Criterion (AIC) and Bayesian Information Criterion (BIC), with lower values indicating improved fit. Statistical significance was defined at *P* ≤ 0.05. All analyses were conducted using Stata version 18 (College Station, TX, USA).

## Results

### Labor characteristics and workforce demographic summary

#### Farm-level employment of TN-visa workers

Table 1 summarizes the distribution and utilization of TN-visa workers across surveyed swine production systems in six U.S. states. The data show the number of TN-visa employees relative to total hired labor at the farm level, enabling calculation of farm-specific reliance on TN-visa labor.

**Table 1:** Distribution and utilization rates of TN-visa workers across surveyed swine farms.

State^a^	Company^b^	Farm^b^	Total TN-visa employees	Total hired employees	Percentage (%)^c^
***MO***	1	A	9	12	75.0
***MO***	2	A	3	7	42.9
***MO***	3	A	5	17	29.4
***MO***	3	B	11	19	57.9
***MO***	3	C	12	19	63.2
***MO***	3	D	8	18	44.4
***MO***	3	E	10	19	52.6
***MO***	4	A	11	13	84.6
***MO***	4	B	10	12	83.3
***IA***	1	A	13	22	59.1
***IN***	1	A	3	7	42.9
***IL***	1	A	11	36	30.6
***IL***	1	B	9	21	42.9
***IL***	2	A	2	2	100.0
***OH***	1	NA*	14	210	6.7
***OH***	2	NA*	7	24	30.0
***NC***	1	A	28	36	77.8
***NC***	1	B	7	11	63.6
***NC***	1	C	10	14	71.4
***NC***	1	D	7	7	100.0
***NC***	1	E	6	11	54.5
***NC***	1	F	2	5	40.0
***NC***	1	G	9	11	81.8
***NC***	1	H	7	11	63.6
***NC***	1	I	7	11	63.6
***NC***	1	J	4	7	57.1
***NC***	1	K	4	7	57.1
***NC***	1	L	3	7	42.9
***NC***	1	M	**9**	12	**75.0**
***NC***	2	A	9	17	52.9
***NC***	2	B	7	12	58.3
***NC***	2	C	4	12	33.3
***Total***	12	30	**261**	649	**40.2**

a Percentage of total hired labor; IA, Iowa; IL, Illinois; IN, Indiana; MN, Minnesota; MO, Missouri; NC, North Carolina; OH, Ohio in participating farms.

b Farm and company identifiers are anonymized.

c Percentage calculated as TN-visa employees divided by the total hired employees.

* NA indicates that farm identifiers were not available or not collected.

Thirty surveyed farms employed 261 TN-visa workers among a total of 649 hired employees, representing a combined utilization rate of roughly 40%. Utilization varied widely across farms, ranging from 7% in one Ohio operation to 100% in several farms in Illinois and North Carolina. At the state level, average TN-visa utilization among participating farms was 64% in North Carolina, 59% in Iowa, 58% in Missouri, 43% in Indiana, 37% in Illinois, and 9% in Ohio.

#### Responses per state and type of survey

In total, 261 responses were obtained—211 in-person and 50 online surveys. Respondents were based in 15 states—6 states were surveyed in person and 9 states through the online tool (Table 2). The targeted response rate from the original six states was not met in Illinois, Iowa, and Minnesota mainly because of issues associated with in-person recruitment of TN-visa workers, including employers not willing to facilitate recruitment, busy schedules from management and employees, and even biosecurity concerns. However, additional surveys were conducted in North Carolina, Missouri, and Ohio to compensate for the low response rates in those states.

**Table 2:** **Number of responders (TN-visa workers) per survey type by state (*n*** = **261).**

State	In person^a^	Online^b^	Total responses per state	Percentage (%)^c^
***North Carolina***	85	5	90	34.5
***Missouri***	56	8	64	24.5
***Ohio***	32	2	34	13.0
***Iowa***	16	5	21	8.0
***Illinois***	14	5	19	7.3
***Indiana***	6	1	7	2.7
***Minnesota***	2	5	7	2.7
***Wyoming***	0	5	5	1.9
***Nebraska***	0	4	4	1.5
***South Dakota***	0	4	4	1.1
***Oklahoma***	0	2	2	0.8
***Pennsylvania***	0	2	2	0.8
***Colorado***	0	1	1	0.4
***Kansas***	0	1	1	0.4
***Texas***	0	1	1	0.4
***Total***	**211**	**50**	**261**	**100.0**

a “In-person” surveys were administered face to face with the TN-visa employees in a neutral location.

b “Online” surveys were completed via Qualtrics.

c Percentage calculated as total responses per state divided by the total overall responses in the project.

#### Participant demographics

Most respondents were in their late twenties to early thirties (approximately 60%) and identified as male (approximately 65%). In addition, approximately half reported being raised in a small town or rural area. Two-thirds of the respondents reported prior agricultural experience in row crops and livestock sectors such as dairy and beef cattle, sheep or goats, and poultry. Only a small proportion of respondents reported prior experience specifically in swine production (Table 3).

**Table 3:** Demographic characteristics, educational background, origin, experience, and self-assessment English literacy of TN-visa workers in the TN visa program in the U.S. swine industry survey.

***Age (*n* = 260)***		***n* (%)^a^**
*** 18–25***		5 (1.9)
*** 26–34***		153 (58.8)
*** 35–44***		75 (28.8)
*** 45–54***		21 (8.1)
*** 55–64***		6 (2.3)
***Sex (*n* = 259)***		***n* (%)^a^**
*** Male***		165 (63.7)
*** Female***		94 (36.3)
***Area raised (*n* = 260)***		***n* (%)^a^**
*** City (>100,000 citizens)***		63 (24.2)
*** Suburban/Large town (20,000–100,000 citizens)***		51 (19.6)
*** Small town (5,000–20,000 citizens)***		82 (31.6)
*** Rural area (<5,000 citizens)***		64 (24.6)
***Did you obtain experience in Mexico? (*n* = 261)***		***n* (%)^a^**
*** Yes***		174 (66.7)
*** No***		80 (30.6)
*** Did not answer***		7 (2.7)
***Type of experience***		***n* (%)^b^**
*** Crops***		58 (22.2)
*** Dairy cattle***		55 (21.1)
*** Beef cattle***		44 (16.9)
*** Sheep or goats***		42 (16.1)
*** Poultry***		37 (14.2)
*** Greenhouse***		32 (12.3)
*** Equine***		26 (10.0)
*** Small vet clinic***		11 (4.2)
*** Agricultural sales***		10 (3.8)
*** Packing plant***		10 (3.8)
*** Government***		7 (2.7)
*** Laboratory work***		4 (1.5)
*** Swine***		4 (1.5)
*** Other^c^***		9 (3.6)
***Highest attained education (*n* = 261)***		***n* (%)^a^**
*** Bachelor’s degree***		226 (86.6)
*** Certification (higher than bachelor level)***		4 (1.5)
*** Specialization degree***		4 (1.5)
*** Master’s degree***		26 (10.0)
*** Doctorate***		1 (0.4)
***Degree earned (*n* = 261)***		***n* (%)^a^**
*** Agronomy***		106 (40.6)
*** Animal Science***		57 (21.8)
*** Veterinary Medicine***		95 (36.4)
*** Biology/Ecology***		18 (6.9)
*** Other^c^***		10 (3.8)
***Preferred language speaking in? (*n* = 261)***		***n* (%)^a^**
*** English***		14 (5.4)
*** Spanish***		210 (80.5)
*** Native dialect***		4 (1.5)
*** Preferred not to say***		33 (12.6)
***Ability to Speak English (*n* = 261)***		***n* (%)^a^**
*** Basic (few words/phrases)***		168 (64.9)
*** Intermediate (relatively fluent)***		78 (30.1)
*** Advanced (full conversations)***		13 (5.0)

a Percentage calculated as total responses per demographic or characteristic cluster divided by the total overall responses in each demographic or characteristic.

b Respondents could select more than one type of prior experience, therefore totals exceed 100%.

c “Other” includes low-frequency job categories.

Regarding educational background, most respondents reported holding at least a bachelor’s degree, consistent with the educational requirements associated with TN-visa eligibility. Because participants were asked to select only their highest level of education, individuals with post-baccalaureate credentials (e.g., certification, specialization, or graduate degrees) reported those qualifications rather than indicating both a bachelor’s degree and a higher credential. This may account for minor inconsistencies in the distribution of reported educational attainment (Table 3). Field-of-study responses further indicated that many participants had training in agronomy, veterinary medicine, and other agriculture-related disciplines.

Regarding self-assessment of English literacy, over 80% of respondents indicated that they preferred conversing in Spanish. Approximately 95% of the participants decided to fill out the survey in Spanish, even though they were offered an English version (Table 3).

In terms of length of employment under a TN-visa program, just over half were in it between 3 and 6 years. Additionally, when asked about the swine farms where they were currently working and the type of roles they were playing on those farms, most respondents indicated they worked in the farrowing area and on farms with a herd size larger than 2,500 head (82%). A minority worked in the breeding and gestation areas. Most TN-visa workers held hourly positions, while a smaller portion were employed in leadership roles, including department leads, managers, and trainees preparing for management positions (Table 4).

**Table 4:** Employment and farm characteristics of TN-visa workers in the U.S. swine industry.

***TN visa participation^a^ (*n = *261)***	**n (%)^b^**
*** <3 years***	102 (39.1)
*** 3–6 years***	138 (52.9)
*** >6 years***	21 (8.0)
***Farm herd size (*n = *257)***	**n (%)^b^**
*** <1000 heads***	5 (1.9)
*** 1001–2500 heads***	42 (16.3)
*** 2501–5000 heads***	101 (39.3)
*** >5000 heads***	109 (42.4)
***Farm position (*n = *261)***	**n (%)^b^**
*** Farm manager***	13 (5.0)
*** Production manager trainee***	15 (5.8)
*** Team lead***	37 (14.2)
*** Hourly employee***	192 (73.5)
*** Other***	4 (1.5)
***Working production stage^c^ (*n = *261)***	**n (%)^b^**
*** Farrowing***	159 (60.9)
*** Breeding***	89 (34.1)
*** Gestation***	84 (32.2)
*** Gilt Development Unit***	41 (15.7)
*** Nursery***	16 (6.1)
*** Finisher***	13 (4.9)
*** Boar stud***	1 (0.4)
*** Other^d^***	6 (2.3)

a TN-visa participation clusters are mutually exclusive.

b Percentage calculated as total responses per cluster divided by the total overall responses.

c Respondents could select more than one type of prior experience; totals exceed 100%.

d “Other” includes low frequency production stages.

#### Motivation, professional and personal goals

**Figure 1:**
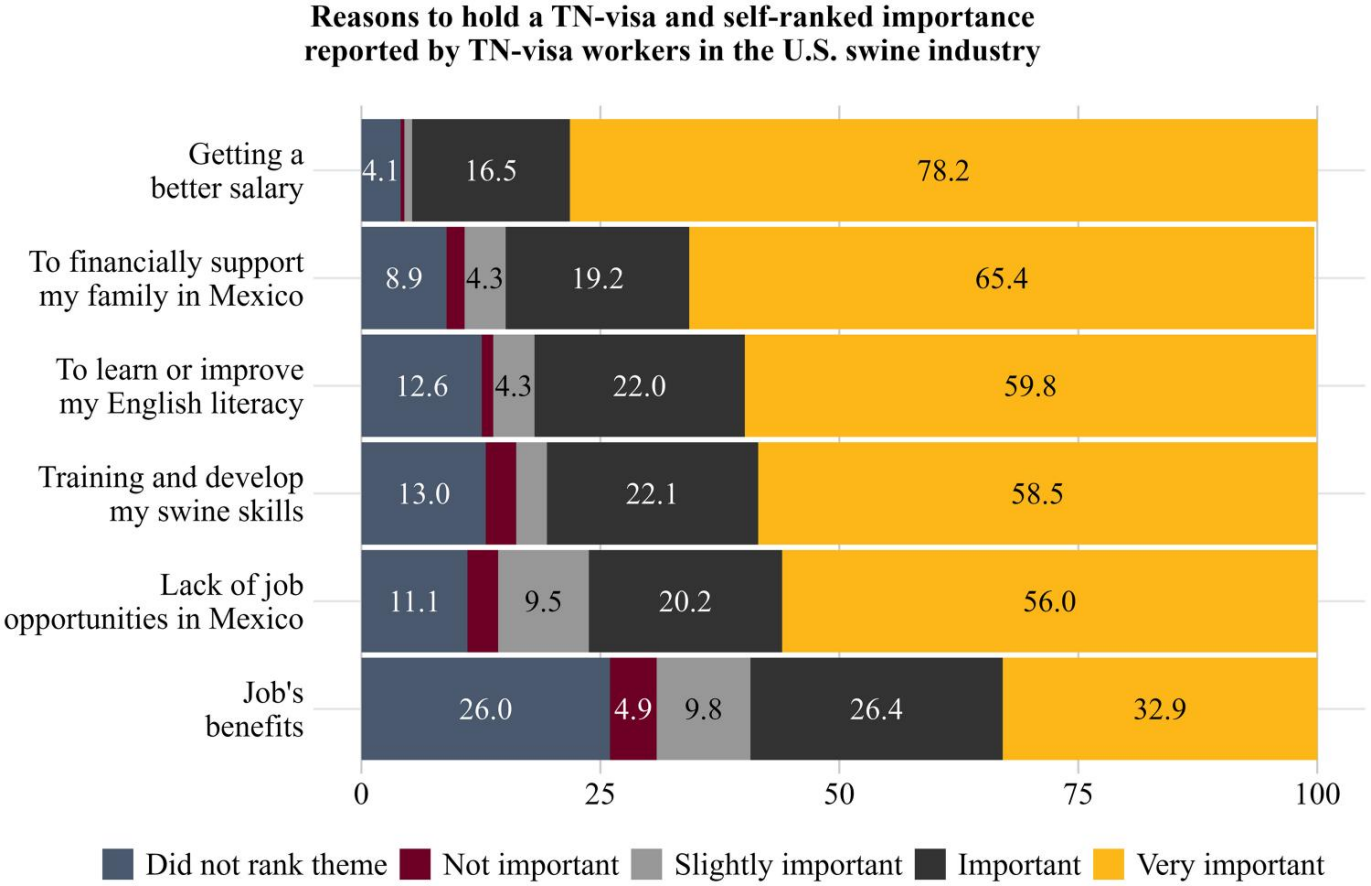
Reasons to hold a TN visa and self-rated importance reported by TN-visa workers in the U.S. swine industry (Percentages less than 4% not shown. Multiple answers per respondent were possible.).

Figure 1 respondents’ ratings of the importance of different reasons for working in the United States under a TN visa. “Getting a better salary” was the most frequently rated as very important, followed by “financially supporting my family in Mexico,” “learning or improving English,” “developing swine skills,” and “lack of job opportunities in Mexico.” By contrast, employment benefits were less frequently rated as very important relative to these other motivations.

**Figure 2:**
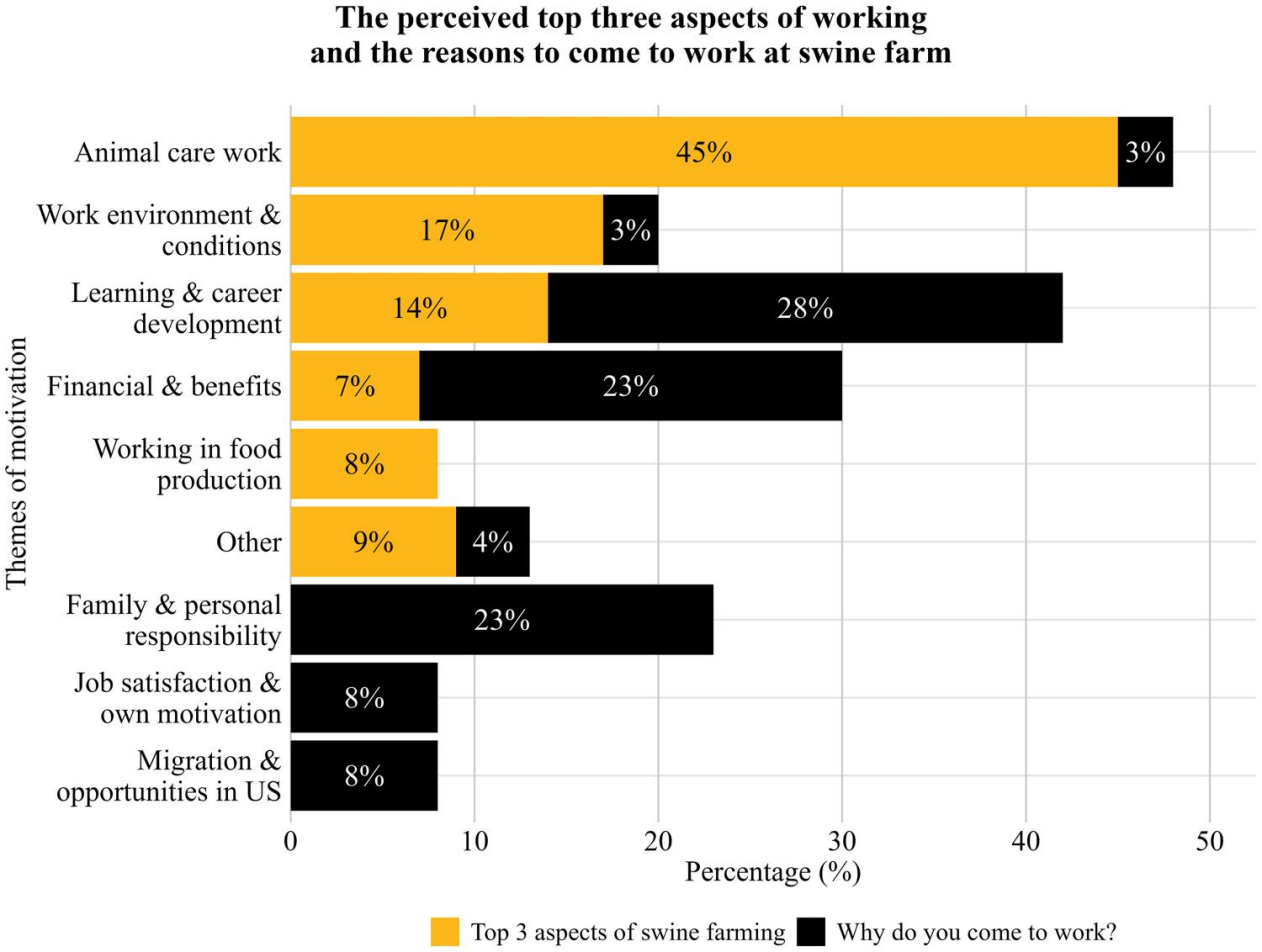
The perceived top three aspects of working at a swine farm and the reasons to come to work every day by TN-visa workers in the U.S. swine industry.

The data shown in Fig. 2 illustrate TN-visa workers’ themes of motivation. Respondents were asked two open-ended questions to assess their motivation for taking their current job. First, they were asked, *“What are the top three aspects about working at a swine farm?”*. Respondents were also asked, “Why do you come to work at the farm every day?”.

When asked to identify the top three aspects they valued most about working at a swine farm, animal-related tasks clearly dominate these responses. Work environment and opportunities for learning and career development also emerge as important job characteristics. Conversely, when looking at motivation to work, the emphasis shifts toward family obligations, financial needs, and personal responsibilities. Career growth remains a strong motivator, while job satisfaction and opportunities in the U.S. also play a role, but to a lesser extent.

**Figure 3:**
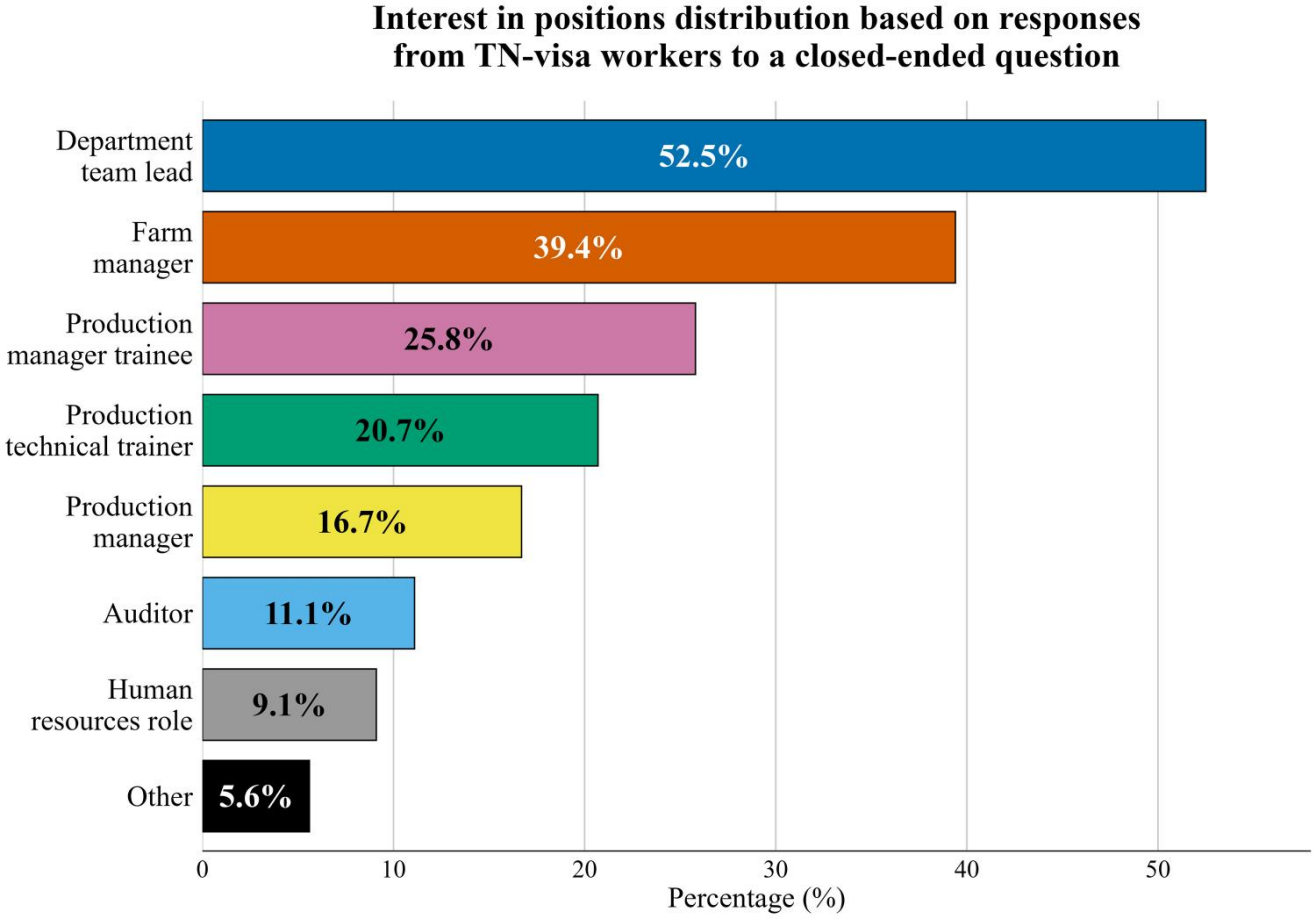
Interest in positions distribution based on responses from TN-visa workers to a closed-ended survey question (Percentage exceeds 100% because participants were allowed to select multiple response.).

Figure 3 exhibits respondents’ most frequently expressed interest in positions involving departmental leadership or farm management. Opportunities related to managerial training and technical training also attracted considerable attention, though to a lesser extent. Interest in roles such as production management, human resources, auditing, or other miscellaneous positions was present but represented a smaller share of the overall responses.

**Figure 4:**
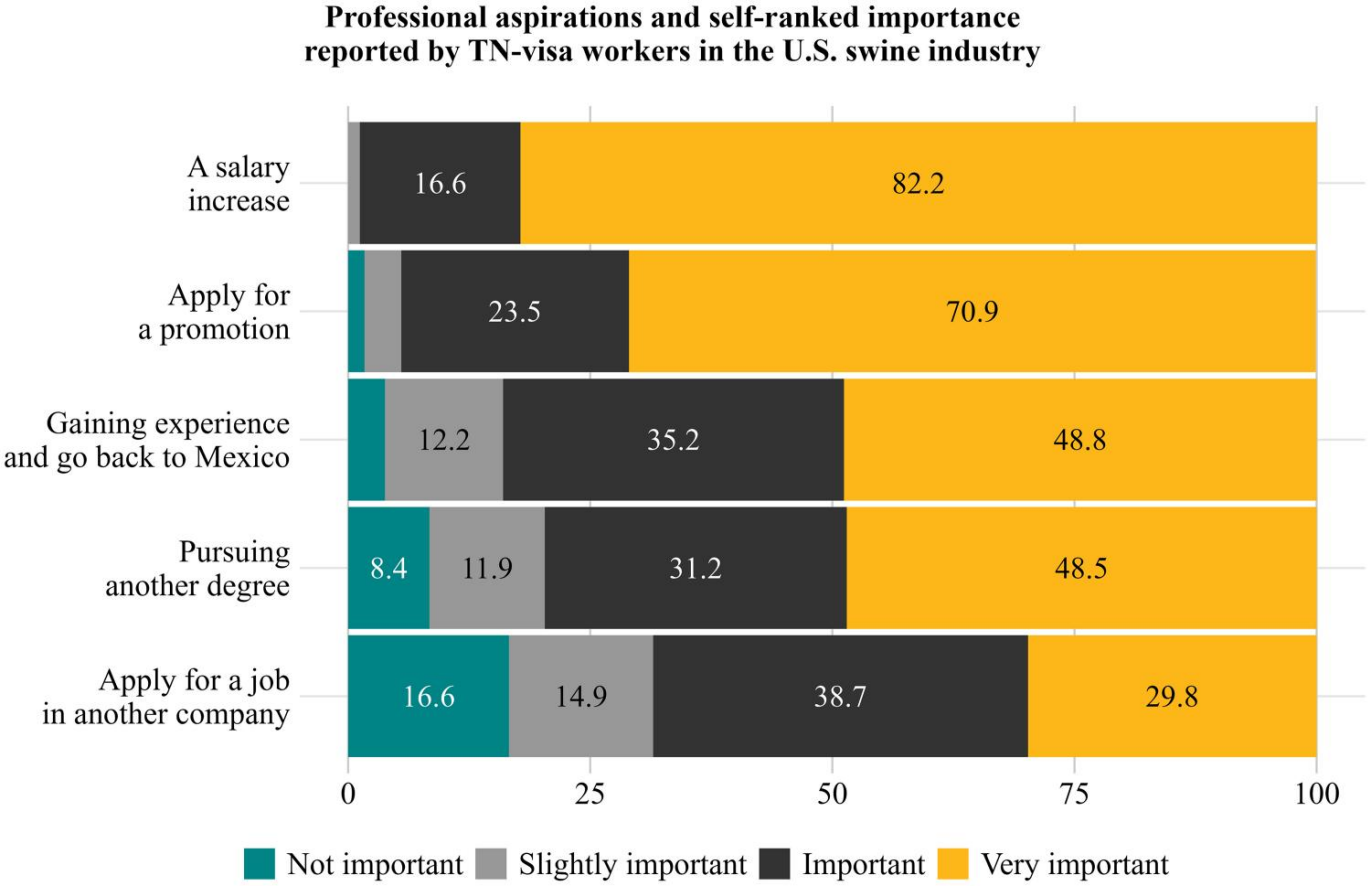
Professional aspirations and self-ranked importance reported by TN-visa workers in the U.S. swine industry (Percentages less than 4% not shown. Multiple answers per respondent were possible.).

The data displayed in Fig. 4 depicts TN-visa workers’ self-assessed importance of various professional aspirations. Each bar corresponds to a distinct theme, with color segments representing the relative frequency of ranked importance levels. Additionally, this figure indicates that respondents placed substantial importance on financial advancement and career progression during their time in the United States. Many also viewed their current employment as a means of gaining experience before either returning to Mexico or pursuing further education. Seeking employment at another company was comparatively less frequently endorsed, as many respondents prioritized financial advancement and career progression over changing jobs.

**Figure 5:**
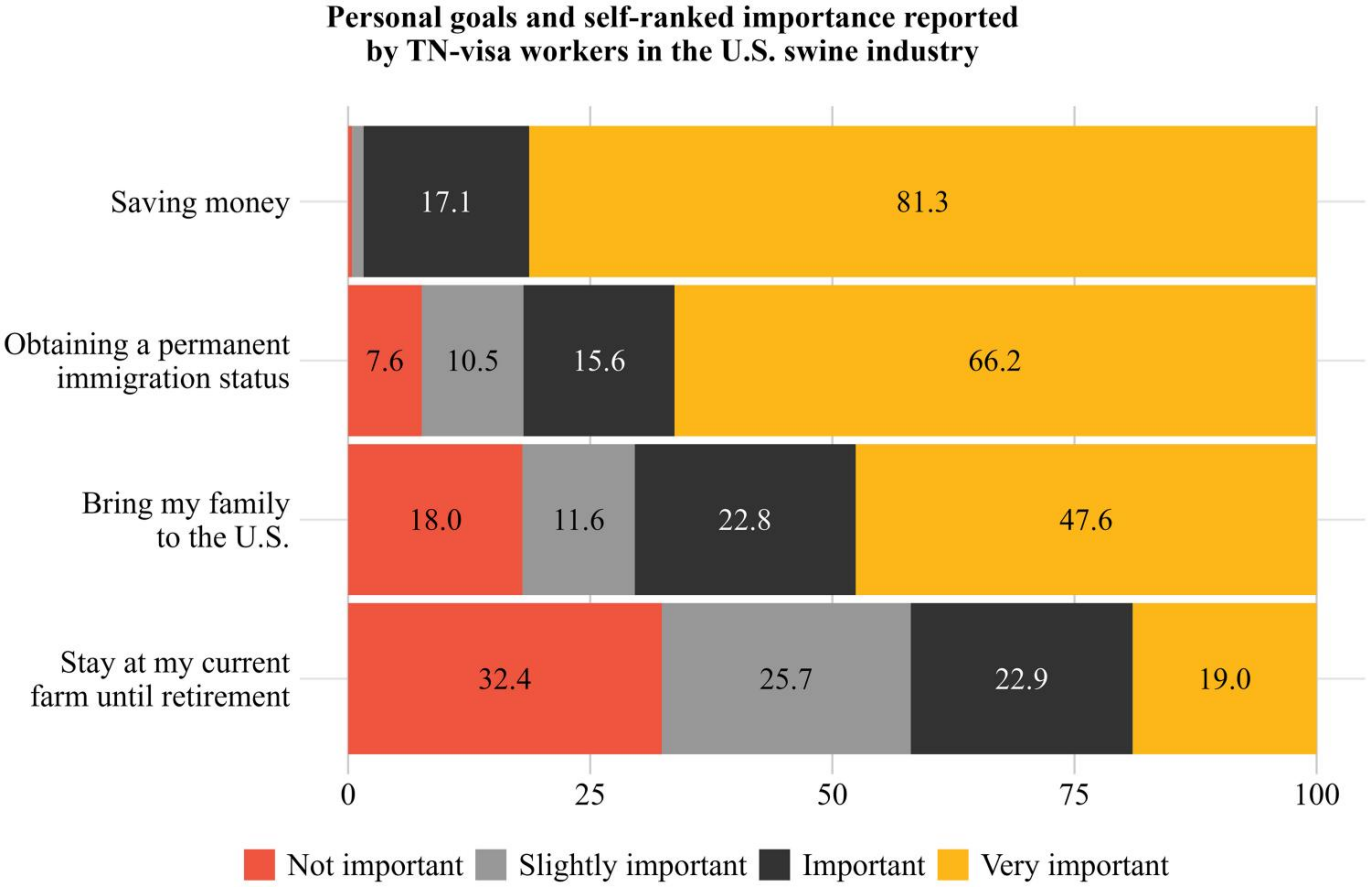
Personal goals and self-ranked importance reported by TN-visa workers in the U.S. swine industry (Percentages less than 4% not shown. Multiple answers per respondent were possible.).

Figure 5 shows TN-visa workers’ self-assessed importance of various personal goals. The results reveal that saving funds and securing long-term immigration stability are the most highly prioritized aspirations among respondents. A considerable proportion also emphasized family reunification in the U.S. as an essential aim. In contrast, remaining at the current farm until retirement was the least frequently endorsed personal goal.

**Figure 6:**
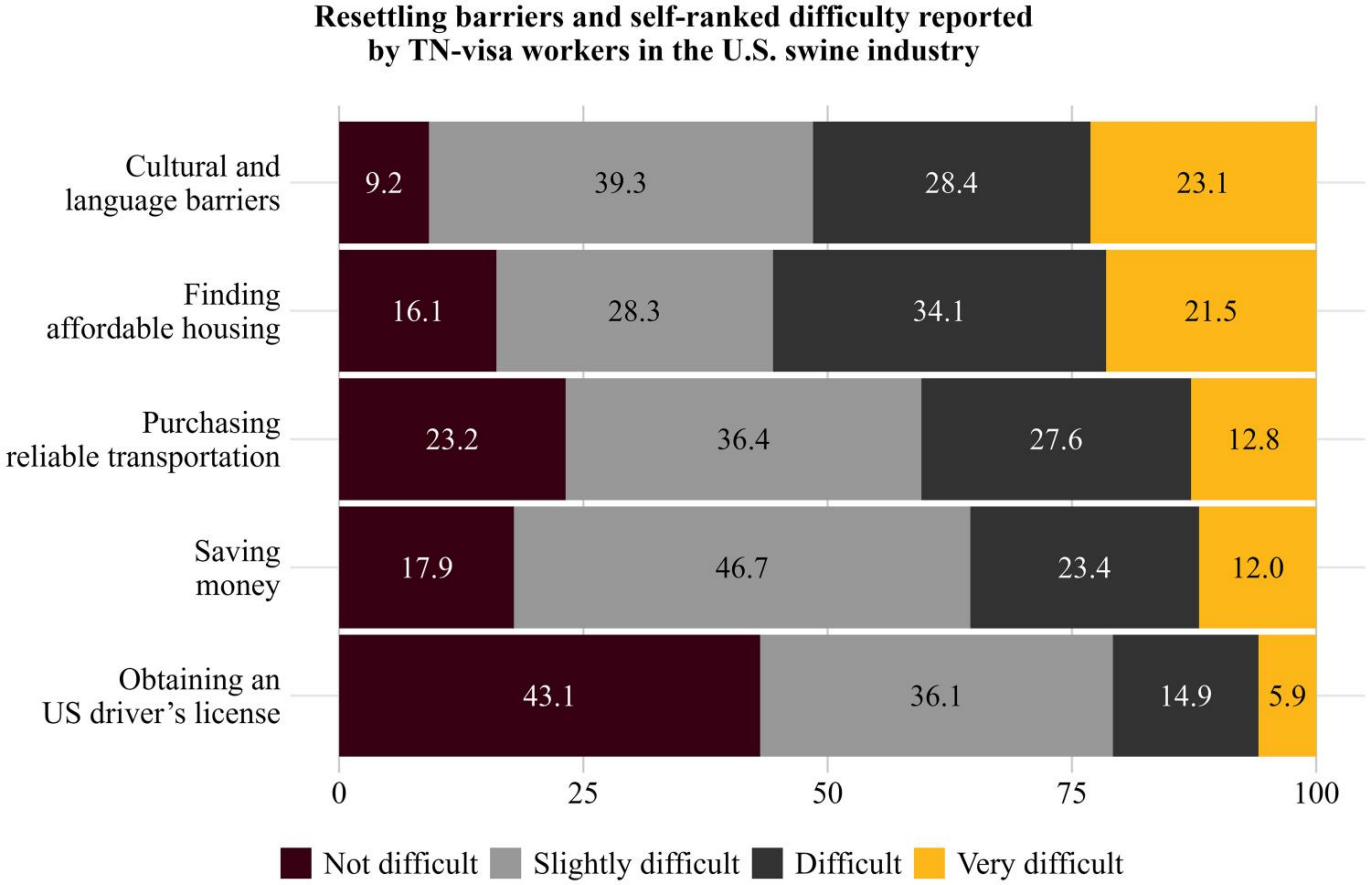
Resettling barriers and self-ranked importance reported by TN-visa workers in the U.S. swine industry.

The next figure illustrates the degree of perceived difficulty that TN visa workers experience with various resettlement challenges when moving to the U.S. (Fig. 6). Among resettlement challenges, respondents most frequently rated cultural adaptation (including language adjustment) and finding affordable housing as moderately to highly difficult. Transportation, conversely, showed a lower frequency of responses being “very difficult” compared with the first two themes. For the theme “saving money” and “obtaining a U.S. driver’s license” a little less than half of respondents classified their experience as “slightly difficult” and “not difficult” correspondingly.

The survey captured TN-visa workers’ awareness of promotion; 59.8% of them were aware of promotions, with 25.1% of respondents indicating they were not interested in seeking a promotion and 13.1% reporting that they were not sure about pursuing career advancement. Finally, 1.9% of TN-visa employees did not answer this question.

On the other hand, regarding TN-visa workers interested in promotions, the majority of respondents (75.9%) indicated affirmative interest, suggesting strong aspirations for upward mobility within their current employment context. Meanwhile, 11.1% and 12.3% reported not being interested in pursuing a promotion or being unsure about it, respectively. Furthermore, there were only 0.8% non-responses to this question.

#### Predictors of career advancement and role attainment

The final multivariable mixed-effects logistic regression model included the number of years in the TN visa program, previous agriculture experience (other than swine), sex, degree, and promotion opportunity. Sex and previous agriculture experience apart from swine were retained as confounding variables.

The results presented in Table 5 suggest that the best predictors of a TN-visa worker’s being in a management position were years in the TN visa program and promotion opportunity awareness. Compared with workers who had been in the TN-visa program for fewer than 3 years, respondents with 3–6 years and more than 6 years in the program had approximately three-fold (OR = 3.15, *P* = 0.005) and four-fold (OR = 4.00, *P* = 0.014) higher odds, respectively, of holding a managerial role. Workers who reported being aware of promotion opportunities at their workplace also had more than four-fold higher odds of being in upper management compared with those who were not aware of such opportunities (OR = 4.4, *P* = 0.003; Table 5). Female TN-visa workers tended to have two-fold higher odds of being placed in upper-level management as compared to their male counterparts (OR = 2.00; *P* = 0.065). Previous work experience and educational degree were kept as confounders in the final model. No other significant results were found.

**Table 5:** Results from a multivariable mixed effects ordered logistic regression model^a^ investigating predictors for U.S TN-visa workers hired in upper management roles.

Variable	Category	Odds Ratio	SE	95% CI	*P*-Value
***Years in the TN-Visa program***	<3	Reference^b^			
	3–6	3.15	1.30	1.41–7.07	0.005
	>6	4.00	2.26	1.32–12.1	0.014
***Previous agricultural work experience other than swine***	Crops/greenhouse	Reference			
	Equine, large or small ruminants	1.33	1.19	0.23–7.69	0.75
	Poultry	0.47	0.62	0.04–6.14	0.57
	Multi-agricultural	0.93	0.61	0.26–3.33	0.91
	Other	0.76	0.62	0.15–3.73	0.73
	No previous work experience	0.51	0.44	0.14–1.88	0.31
***Sex***	Male	Reference			
	Female	2.00	0.75	0.96–4.19	0.065
***Degree***	Agronomy	Reference			
	Animal science	1.79	0.87	0.67–4.66	0.25
	Veterinary medicine	0.68	0.34	0.25–1.83	0.44
	Biology/ecology	1.06	0.76	0.26–4.33	0.93
***Promotion opportunity awareness***	No	Reference			
	Yes	4.40	2.19	1.65–11.7	0.003

a Multivariable mixed-effects model with ordered logistic regression including respondent home state as a random effect. Statistical significance was declared at *P* ≤ 0.05.

b Reference = baseline category for comparison within each variable.

## Discussion

One of the most significant threats to the long-term sustainability of the U.S. swine industry is the ongoing shortage of a reliable and skilled workforce. This challenge has been driven by factors such as rural depopulation, declining interest among U.S.-born workers in agricultural employment, and demographic shifts within the domestic labor force. In response to these constraints, many swine producers have increasingly relied on the TN visa program, established under the North American Free Trade Agreement (NAFTA), to recruit highly educated professionals—predominantly from Mexico—who provide year-round labor and bring substantial technical training and expertise. Despite the advantages associated with this workforce, high turnover among TN-visa employees remains a persistent concern, potentially undermining workforce stability and long-term operational efficiency. Accordingly, the objectives of this study were to anonymously assess the demographic characteristics, cultural and professional backgrounds, prior work experiences, and motivations of TN-visa workers employed in major U.S. pork-producing states. Additionally, this study aimed to identify key factors associated with career advancement and role attainment among TN-visa workers within the swine industry.

One of the clearest findings of this study is that TN-visa workers in swine production do not resemble a generic replacement labor pool. Respondents reported substantial prior agricultural experience (particularly in crops and other livestock sectors), and most held formal postsecondary training in agriculture-related fields. In this sense, the TN-visa workforce appears to enter swine production with significant technical preparation, even when prior direct experience in swine itself is limited. This distinction matters because it suggests that many of these workers arrive not as unskilled entrants, but as trained migrant professionals whose expertise may be only partially translated into recognized authority or specialized responsibility within the farm setting. The findings therefore point to a potential mismatch between the qualifications workers bring and the roles they initially occupy, raising broader questions about how migrant expertise is assessed, utilized, and rewarded in agricultural labor systems.

The demographic profile of respondents indicates that a significant number of TN-visa workers commence their careers in U.S. swine production at an early or mid-career stage. Most surveyed workers were in their late twenties and early thirties, a pattern that is broadly consistent with prior work indicating that TN-visa professionals from Mexico are younger on average than both native-born workers and other non-TN skilled immigrants (Shih 2024). Rather than treating age as a standalone explanatory factor, however, it is more useful to interpret this finding in relation to career formation: workers at this stage may be especially attentive to opportunities for skill development, leadership growth, and future mobility. This interpretation is consistent with the present data, in which respondents expressed strong interest in promotion, management, and technical advancement. It also suggests that retention efforts may be more effective when framed not only around immediate productivity but also around longer-term professional development.

Approximately 64% of respondents in our sample identified as male, a sex distribution broadly consistent with prior research involving Hispanic swine farmworkers, mainly of Mexican origin, in which male workers also represented the majority of the labor force (Acevedo-León et al. 2024). Although sex was not a central focus of this study, prior work suggests that female workers may encounter distinct challenges in livestock production settings, such as limited access to training and resources compared to their male counterparts. Nevertheless, in addition to the significance of women in swine farms, the authors want to emphasize the insufficient attention devoted to sex equality. As such, future research should continue to examine how gender may influence work assignments, advancement opportunities, and experiences of inclusion within swine production systems.

While financial factors were pivotal in workers’ decisions to accept swine jobs in the U.S., the results complicate any simplistic economic interpretation of TN-visa participation. Respondents emphasized better wages, remittance-related obligations, and the desire to support family members in Mexico as major motivators, which is consistent with broader scholarship on Mexican labor migration that highlights economic mobility, family obligation, and transnational financial support as key drivers (Pew Research Center 2015; Berg et al. 2025; Franco 2025). Vaughan (2004) similarly argued that the appeal of the TN visa for highly skilled workers is partly shaped by the wage differential between the U.S. and Mexico. At the same time, workers in the present study also identified skill development, professional growth, and everyday workplace climate as meaningful dimensions of their employment experience. In particular, the prominence of a “good work environment” as a recurring motivator suggests that retention is affected not only by income but also by the social and relational conditions of work.

This finding is especially important because it shifts the conversation from labor recruitment to workplace incorporation. The significance respondents placed on the work environment suggests that long-term engagement in swine production may depend on whether workers experience the farm as a site of respect, predictability, and professional legitimacy rather than solely as a site of wage labor. This interpretation is consistent with prior research in agricultural settings showing that linguistic and cultural differences do not necessarily produce mistrust or conflict in and of themselves; rather, managerial disposition, interpersonal respect, and the quality of supervisory relationships often play a larger role in shaping workplace satisfaction and retention (Clift 2021). In this sense, a “positive work environment” should be understood as a fundamental organizational factor that plays a decisive role in retaining skilled migrant workers, rather than as a secondary or merely “feel-good” consideration.

Language remains especially important in this regard. Most respondents reported greater comfort communicating in Spanish, and the majority completed the survey in Spanish when given the option, despite being offered an English-language version. Rather than treating this as surprising, it is more analytically useful to understand it as evidence that formal eligibility for skilled migration does not necessarily translate into ease of communication within English-dominant workplaces. For TN-visa workers, language is not simply a matter of translation; it models access to training, the ability to interpret instructions, confidence in voicing concerns, and the visibility of one’s competence to supervisors. Language may, therefore, shape not only task performance but also access to training, confidence in communication, and perceived opportunities for advancement. In this sense, Spanish-available materials and bilingual supervisory practices, through technological tools, should not be viewed as optional accommodations but as central components of workforce development and organizational inclusion.

The present findings also suggest that promotion should be understood as an organizationally structured process rather than a purely individual outcome. Respondents expressed strong interest in leadership, management, and technical growth, indicating that many view their current positions not as fixed endpoints but as part of a broader professional trajectory. This is particularly important in light of the educational and agricultural experience many respondents reported bringing with them into the U.S. swine sector. When workers enter the industry with substantial credentials and prior experience yet remain concentrated in hourly or non-managerial roles, aspirations for advancement become an important lens through which to understand retention. Workers may be willing to remain in demanding production environments when they can identify a credible pathway toward increased responsibility, recognition, and future mobility.

The regression analysis, while secondary to the broader descriptive findings, supports this interpretation. Workers with longer tenure in the TN-visa program and greater awareness of promotion opportunities were more likely to hold management roles. It can be intuitive that time in role, experience, and knowledge of advancement pathways matter. However, their significance in this study lies less in the individual predictors themselves than in what they reveal about workplace structure. Advancement appears to depend not only on worker motivation or qualifications already possessed but also on whether opportunities are visible, legible, and institutionally supported within the farm. Put differently, professional mobility appears to be shaped by organizational transparency and continuity as much as by individual ambition. This interpretation is especially relevant in a labor segment where workers are formally skilled but may not always enter with immediate access to the kinds of social, linguistic, or organizational capital that make advancement pathways easy to navigate.

These outcomes have practical implications for retention. In production systems where the number of formal upper-management positions is necessarily limited, workers’ interest in advancement may still be supported through structured developmental assignments that expand leadership exposure and specialized responsibility without requiring an immediate title change or full managerial vacancy. Examples might include onboarding support, animal welfare coordination, safety leadership, or feed inventory oversight. When paired with formal recognition and, where feasible, monetary or non-monetary incentives, such assignments can create more credible intermediate pathways between routine production labor and upper-level management. These arrangements may also help workers build a documented record of leadership and technical specialization that is legible within the current operation and transferable across employers. In this sense, workforce development in swine production should not be reduced to one-time training events but should be understood as the deliberate creation of visible and meaningful progression within the workplace.

The educational profile of respondents reinforces this point. Consistent with prior research on TN-visa professionals (Shih 2024), workers in the present study were highly educated relative to many other immigrant agricultural labor populations. This does not mean, though, that all workers require the same kind of training. On the contrary, the diversity of prior educational backgrounds and agricultural experiences reported here suggests that swine operations may benefit from tiered and differentiated approaches to workforce development. Rather than assuming a uniform starting point, employers, extension personnel, and industry stakeholders may need to design training systems that recognize prior expertise while also supporting adaptation to the specific technical, linguistic, and organizational demands of U.S. swine production. Such an approach is more likely to treat training as a process of professional integration than as simple remediation.

The reported themes on resettlement barriers further underscore that retention unfolds within broader conditions of temporary labor migration. Respondents identified language and housing as notable challenges when adjusting to life in the United States, suggesting that integration into farm work is inseparable from the practical demands of settling into everyday life. Prior research on Hispanic immigrants has similarly shown that even when workers are legally present and occupationally authorized, language barriers, social isolation, and structural stressors can complicate adjustment and affect well-being (Ramos et al. 2015; Santana 2024). These challenges extend beyond conditions external to employment and actively outline the pace at which farm routines are learned, the degree of confidence exercised in interactions with supervisors and coworkers, and the longer-term sustainability of work engagement. Thus, retention should not be understood merely as an employer-side management issue but as a process affected by the interaction of workplace conditions and wider resettlement demands.

The finding that many respondents prioritized long-term immigration stability as a personal goal should also be interpreted carefully. This study is not designed to evaluate immigration policy pathways, and it does not make legal claims regarding transition mechanisms within or beyond TN status. Yet the weight of long-term stability in workers’ responses suggests that employment decisions are being made within a broader horizon of uncertainty about continuity, family planning, and future settlement. For workers employed under a temporary visa framework, aspirations for permanence or greater stability may shape how they evaluate current employment conditions, advancement opportunities, and long-term commitment to a particular employer or sector. In this sense, retention is not only about whether workers are satisfied in the present, but also about whether the workplace offers a plausible basis for imagining continuity and professional growth over time. Moreover, possible TN visa restrictions through NAFTA renegotiation and increased compliance checks can create uncertainty for this program (Salamanca-Pacheco 2018). Hence, securing a permanent immigration status is important for the employees to be able to stay indefinitely with their current employer without being concerned about a visa renewal and planning the future of their personal lives.

A final point concerns worker voice and organizational hierarchy. The present study did not directly measure communication dynamics with supervisors, and therefore any interpretation here must remain thoughtful. Nonetheless, the combination of strong Spanish preference, interest in advancement, and the importance placed on work environment suggests that communication should be treated as more than a technical matter. In settings where workers depend on employers for ongoing visa-linked employment, where supervisory authority is embedded in highly hierarchical production systems, and where linguistic asymmetries remain pronounced, the ability to ask questions, express disagreement, or articulate career goals may be unevenly distributed. For that reason, future research should examine more directly how language, supervisory culture, and visa-linked dependency interact to influence worker voice, trust, and perceived access to opportunity in swine operations.

Thus, the utmost analytically significant contribution of this study is that TN-visa workers in swine production constitute a highly educated, occupationally ambitious, and organizationally consequential workforce whose retention depends on how farms structure recognition, communication, and credible mobility. The most important question, therefore, is not simply whether these workers can help fill labor shortages, but whether the industry is prepared to move beyond labor substitution and invest in workforce development practices that more fully recognize their expertise, aspirations, and long-term importance to the sustainability of U.S. swine production.

## Conclusion

This study highlights a central tension in the position of TN-visa workers within U.S. swine production: they are recruited with high levels of education, experience, and with a desirable skill toolbox for swine farms. Over the years, they have proven to be dependable and increasingly indispensable workers; still, their retention is influenced by organizational conditions that extend well beyond compensation alone. While respondents emphasized wages, family support, and financial mobility as major reasons for working in the U.S., they also identified work environment, language accessibility, and opportunities for advancement as key factors shaping their day-to-day experience of employment. These findings suggest that TN-visa workers are not purely responding to labor demand; they are navigating a process of occupational incorporation in which recognition, communication, and future possibility matter alongside income.

The association between management roles, longer tenure in the TN-visa program, and awareness of promotion opportunities further indicate that professional mobility is structured by workplace practices rather than determined solely by worker motivation or qualification. Respondents expressed strong interest in leadership and advancement, yet these aspirations appear to unfold within organizational environments where pathways upward may be unevenly visible or limited in scope. This points to a broader issue of underutilized human capital: a workforce entering the industry with substantial educational preparation and professional ambition may still encounter restricted opportunities to convert those assets into recognized authority, specialized responsibility, or long-term advancement.

For the swine industry, the implication is clear. If TN-visa workers are increasingly central to labor force stability, then retention must be approached as a question of workforce development and organizational inclusion rather than simple labor substitution. Training in Spanish, clear promotion paths, and structured developmental assignments that shift responsibility to leadership and technical specialization are all practical ways to improve retention. More fundamentally, the findings suggest that the future sustainability of swine production depends on whether the industry can move beyond reliance on migrant labor as a stopgap and instead create organizational conditions in which skilled migrant workers are recognized as professionals with legitimate claims to growth, responsibility, and continuity within the sector.

## Limitations

This study should be interpreted in light of several methodological considerations. First, although the sample of 261 TN-visa workers is substantial for an understudied and relatively difficult-to-access workforce, participants were recruited through convenience sampling, and no formal sample size calculation was performed. Accordingly, the findings should not be interpreted as nationally representative of all TN-visa workers in U.S. swine production, particularly those employed in smaller operations or in states not captured through recruitment. Rather, the study is best understood as providing an industry-level, multi-state characterization of a challenging-to-reach labor segment.

Second, data were collected through both in-person and online survey administration. While this approach improved overall reach and allowed participation from workers across a broader geographic area, the two methods of administration differed in ways that may have shaped response patterns, including setting, incentive availability, and the opportunity to request clarification during survey completion. These differences may have introduced unintentional method-related variation in responses and should be considered when interpreting the findings.

Third, because recruitment goals were not met uniformly across all originally targeted states, the study was not designed to support robust state-level comparisons. For this reason, the findings are more appropriately interpreted at the industry level rather than as representative of specific states, companies, or production systems.

Within these bounds, this study offers novel and important insight into the demographic profile, prior experience, motivations, workplace priorities, resettlement challenges, and advancement aspirations of TN-visa workers in the U.S. swine industry—an understudied yet increasingly consequential workforce segment.

## Supplementary Material

txag047_Supplementary_Data
