# Supplementary material for: Recognizing the cultural background, motivation, and experience of TN-visa workers in the U.S. swine industry
Source: Transl Anim Sci. 2026 Apr 22;10:txag047. doi: 10.1093/tas/txag047 (PMC13159992; doi:10.1093/tas/txag047)
Supplement: txag047_Supplementary_Data [file txag047_supplementary_data.zip › S1_TN visa paper Survey English.pdf]

Extension
University of Missouri

Understanding the experiences of TN-visa workers on U.S. swine industry

Background:

You are being invited to take part in a research project. You must be 18 years of age or older. **Your participation is voluntary, your responses are anonymous, and you may stop being in this study at any time.**

The purpose of this research project is to capture **information about the backgrounds and experiences of TN-visa employees on US swine farms**. We are interested in what makes their job fulfilling, identifying the factor(s) that could lead them to leave the farm for other opportunities and identify tools and incentives that might increase their morale and reduce turnover.

You are being asked to fill out a survey. An employee's decision about research participation will not affect (favorably or unfavorably) performance evaluations, career advancement, or other employment-related decisions made by peers or supervisors. Your participation should last up to 30 min. For your time and effort, we will be offering compensation in the amount of $25 (gift card). **The information you provide will be kept confidential and only the research team will have access.**

If you have questions about this study, you can contact Dr. Talita Resende at the Ohio State University at 330-263-8029 or resende.2@osu.edu, or Dr. Magdiel Lopez Soriano at the University of Missouri at 573-445-8375 or mlopezsoriano@missouri.edu. If you have questions about your rights as a research participant, please contact the University of Missouri Institutional Review Board (IRB) at 573-882-3181 or muresearchirb@missouri.edu. The IRB is a group of people who review research studies to make sure the rights and welfare of participants are protected.

You can ask the researcher to provide you with a copy of this consent for your records, or you can save a copy of this consent if it has already been provided to you. We appreciate your consideration to participate in this study.

Q1. Do you consent to taking the following survey?

☐ YES → *please continue* ☐ NO – *please hand the survey back*

Extension
University of Missouri

Q2. What state do you currently work in?

☐ Iowa ☐ Illinois ☐ Minnesota ☐ Missouri ☐ Ohio

☐ Other: ____

2a: what company do you currently work for? ____________________________

Q3. How long have you participated as TN in the United States? *(For example: 5 years + 3 months)*

Years: _______ Months: _______

Q4. Did you work in other industries before coming to work for swine (pigs) both inside and outside the U.S.?

☐ YES → *please continue* ☐ NO – *skip to question 5*

4a. **If yes, which of the following industries did you work in?** *(check all that apply)*

- ☐ Equine
- ☐ Dairy Cattle
- ☐ Beef Cattle
- ☐ Poultry
- ☐ Sheep or Goats
- ☐ Crops
- ☐ Greenhouse
- ☐ Packing Plants
- ☐ Other: ____

Q5. What are the top 3 aspects about working at a swine/pig farm?

1) ____

2) ____

3) ____

Q6. Why do you come to work at the farm every day?

1) ______________________________

2) ______________________________

3) ______________________________

Q7. What is the size of the farm you currently work on (how many pigs)?

☐ 1000 or less ☐ 1001 – 2500 ☐ 2501 – 5000 ☐ 5001 or more

Q8. What is your position at the swine farm?

☐ Hourly employee
☐ Production manager trainee
☐ Head of department or Team lead
☐ Farm manager
☐ Production manager
☐ Other: ______________________________

Q9. In which area of the farm do you spend most of your time? *Check all that apply.*

☐ Farrowing
☐ Breeding
☐ Gestation
☐ GDU
☐ Nursery
☐ Finishing
☐ Other: ______________________________

Extension
University of Missouri

Q10. How satisfied or unsatisfied are you at your current farm?

- ☐ Very Satisfied
- ☐ Satisfied
- ☐ Neither satisfied nor unsatisfied
- ☐ Unsatisfied
- ☐ Very Unsatisfied

Q11. Please explain what makes you satisfied or unsatisfied?

____

____

____

Q12. Have you thought about changing employers from your current one?

☐ YES → *please explain why below* ☐ NO – *skip to question 13*

Q12a. Why were you thinking about changing employers?

____

____

____

Q13. To what extent do you feel your current job is stressful?

- ☐ Not stressful at all
- ☐ Not very stressful
- ☐ Neither very stressful nor a little stressful
- ☐ A little stressful
- ☐ Very stressful

Q13a. If you feel stress, could you explain why?

____

____

____

Extension
University of Missouri

Q14. What benefits do you <u>currently receive</u> as an employee? *(Please click all that apply)*

- ☐ Healthcare
- ☐ Retirement plan
- ☐ Production bonus
- ☐ Attendance in special trainings or conferences
- ☐ Paid vacation (PTO)
- ☐ Referral Bonus
- ☐ Other: ____

Q14a. How important are each of the following benefits <u>you currently receive</u>? *Check the box that best applies to you. If you don't receive a type of benefit – check the circle to the right.*

	**Not important**	**Slightly important**	**Important**	**Very important**	**I Do Not Receive This Benefit**
Healthcare	☐	☐	☐	☐	○
Retirement plan	☐	☐	☐	☐	○
Production bonus	☐	☐	☐	☐	○
Attendance to special trainings or conferences	☐	☐	☐	☐	○
Paid vacation (PTO)	☐	☐	☐	☐	○
Referral bonus	☐	☐	☐	☐	○
Other (specify): ______________ ______________	☐	☐	☐	☐	○

Extension
University of Missouri

Q15. If your employer were to <u>add new benefits</u>, which of the following things would you most like to get? *(check all that apply)*

- ☐ Healthcare
- ☐ Retirement plan
- ☐ Production bonus
- ☐ Attendance in special trainings or conferences
- ☐ Paid vacation (PTO)
- ☐ Referral Bonus
- ☐ Other: ____

Q15a. How important to you are each of the following possible <u>new benefits</u> you might want to receive? *Check the box that best applies to you. If you already receive a type of benefit – check the circle to the right.*

	**Not important**	**Slightly important**	**Important**	**Very important**	**I already receive this benefit**
Healthcare	☐	☐	☐	☐	○
Retirement plan	☐	☐	☐	☐	○
Production bonus	☐	☐	☐	☐	○
Attendance to special trainings or conferences	☐	☐	☐	☐	○
Paid vacation (PTO)	☐	☐	☐	☐	○
Referral bonus	☐	☐	☐	☐	○
Other (specify): ______________ ______________	☐	☐	☐	☐	○

Q16. How important are each of the following reasons to your decision to work in the U.S. on a TN Visa?

	Not important	Slightly important	Important	Very important
To get a better salary	☐	☐	☐	☐
To financially support my family in Mexico	☐	☐	☐	☐
Lack of job opportunities in Mexico	☐	☐	☐	☐
Benefits such as healthcare, retirement	☐	☐	☐	☐
Get training and develop my skills	☐	☐	☐	☐
To learn or improve my English literacy	☐	☐	☐	☐
Other (specify): ____________	☐	☐	☐	☐

Q17. How important are each of the following goals and aspirations to you for your professional career?

	Not important	Slightly important	Important	Very important
A salary increase	☐	☐	☐	☐
Apply for a promotion	☐	☐	☐	☐
Gain experience and go back to Mexico	☐	☐	☐	☐
Apply for a job in another company	☐	☐	☐	☐
Pursue another degree	☐	☐	☐	☐
Other (specify): ____________	☐	☐	☐	☐

Extension
University of Missouri

Q18. How important are each of the following personal goals to you?

	Not important	Slightly important	Important	Very important
Bring my family to the U.S.	☐	☐	☐	☐
Obtaining a permanent immigration status	☐	☐	☐	☐
Stay at my current farm until retirement	☐	☐	☐	☐
To save money	☐	☐	☐	☐
Other (specify): ______________________	☐	☐	☐	☐

Q19. How often do you receive (or participate in) on-the-job training? *(Do not include production meetings)*

☐ Weekly ☐ Monthly ☐ Yearly ☐ Never

☐ Other: ____

Q20. How many training opportunities are you currently receiving from your employer?

____

Q21. How important are each of the following types of potential training opportunities to you?

	Not important	Slightly important	Important	Very important
Developing skills to work with pigs	☐	☐	☐	☐
Employee management	☐	☐	☐	☐
Conflict management	☐	☐	☐	☐
Strategies to support self-care and wellness	☐	☐	☐	☐
Managing personal finances	☐	☐	☐	☐
Other (specify): ______________________	☐	☐	☐	☐

Q22. Are you aware of any opportunities for promotion at work?

☐ **Yes** ➔ *continue* ☐ **No** ➔ *skip to Q24* ☐ **Not sure** ➔ *continue*

Q23. Which of the following types of jobs / opportunities for promotion are available at your farm? *(check all that apply)*

- ☐ Manager trainee on farm
- ☐ Head of department or Team Lead
- ☐ Farm manager on farm
- ☐ Production manager several farms
- ☐ Auditor (animal welfare, safety, environmental)
- ☐ Human resources (Manager, Generalist, Administrative)
- ☐ Production technical trainer
- ☐ Other ____

Q24. If an opportunity comes to apply for a promotion, would you apply?

☐ **Yes** ➔ *skip to Q25* ☐ **No** ➔ *continue* ☐ **Not sure**

Q24a If no, please choose the main reason why from the following *(check one)***:**

- ☐ I do not feel confident about the training received with current employer to apply for a promotion
- ☐ I do not have enough experience to apply for a better position
- ☐ I am not interested in applying for a promotion
- ☐ My application will not be taken in consideration
- ☐ Other: ____

Q25. How difficult are each of the following aspects of settling in or adapting to the U.S.?

	Not difficult	Slightly difficult	Difficult	Very difficult
Cultural and language barriers	☐	☐	☐	☐
Purchasing a reliable transportation	☐	☐	☐	☐
Finding affordable housing	☐	☐	☐	☐
Obtaining a US driver's license	☐	☐	☐	☐
Saving money	☐	☐	☐	☐
Other (specify): ______________________	☐	☐	☐	☐

Q26. What is the highest level of education you have completed or the highest degree you have received?

- ☐ Bachelor's degree
- ☐ Certification (beyond bachelor’s degree)
- ☐ Specialization degree
- ☐ Master's degree
- ☐ Doctorate or professional degree (MD, JD)
- ☐ Other: ____

Q27. What area of study was your degree in?

- ☐ Agronomy
- ☐ Animal Science
- ☐ Veterinary Medicine
- ☐ Biology/Ecology
- ☐ Other: ____

Extension
University of Missouri

Q28. What language are you more comfortable speaking in?

☐ English

☐ Spanish

☐ Native Dialect

☐ Other: ____

Q29. How comfortable are you speaking English?

☐ Basic (only know a few words and phrases)

☐ Intermediate (can speak relatively fluently with others)

☐ Advanced (can have full conversations and feel confident in speaking English)

Q30. When people speak English to you, how much do you understand?

☐ Everything ☐ Most ☐ Some ☐ A little ☐ Very little

Q31. When you speak English, how much do you feel like other people understand?

☐ Everything ☐ Most ☐ Some ☐ A little ☐ Very little

Q32. How frequently are you provided with trainings, Standard Operational Procedures (SOP's), or literature written in Spanish?

☐ All the time ☐ Most of the time ☐ Sometimes ☐ Rarely ☐ Never

Q33. What is your gender or sex?

☐ Male ☐ Female ☐ Prefer not to say

☐ Prefer to self-define: ____

Q34. How old are you?

☐ 18 - 25 ☐ 26 - 34 ☐ 35 - 44 ☐ 45 – 54 ☐ 55 – 64 ☐ 65 or above

☐ Prefer not to say

Q35. How would you describe the area you spent most of your life in?

☐ City (100,000 or more people)

☐ Suburban/Large Town (20,000-100,000)

☐ Small town (5,000 - 20,000)

☐ Rural area (5,000 or less)

Extension
University of Missouri

Q36. **Is there anything else you would like us to know?** *Please provide additional comments here.*

____

____

____

____

____

____

____
