# Supplementary material for: Recognizing the cultural background, motivation, and experience of TN-visa workers in the U.S. swine industry
Source: Transl Anim Sci. 2026 Apr 22;10:txag047. doi: 10.1093/tas/txag047 (PMC13159992; doi:10.1093/tas/txag047)
Supplement: txag047_Supplementary_Data [file txag047_supplementary_data.zip › S2_TN visa paper Survey Spanish.pdf]

Extension
University of Missouri

Entendiendo la experiencia de los trabajadores TN en granjas en la industria porcina de los EE. UU.

Información:

Se le invitan a participar en un proyecto de investigación. Debes tener 18 años o más. **Su participación es voluntaria y puede dejar de participar en este estudio en cualquier momento.**

El propósito de este proyecto de investigación es capturar **sus antecedentes culturales, profesionales y académicos.** Además, es identificar qué hace **que su trabajo sea más satisfactorio,** así como los factores que podrían llevarlo a **dejar la granja en busca de otras oportunidades** e **identificar herramientas e incentivos** que aumentarían su moral y lo mantendrían en la finca como empleado.

Se le pide que complete una encuesta. La decisión de un empleado sobre la participación en esta investigación no afectará (favorable o desfavorablemente) las evaluaciones de desempeño, el avance profesional u otras decisiones relacionadas con el empleo tomadas por sus pares o supervisores. Su participación puede durar hasta 30 min. Por su tiempo y esfuerzo, le ofreceremos una compensación de $25 (tarjeta de regalo). **La información que usted proporcione se mantendrá confidencial y sólo el equipo de investigación tendrá acceso.**

Si tiene preguntas sobre este estudio, puede comunicarse con Dra. Talita Resende de la Universidad Estatal de Ohio al (*330-263-8029 o resende.2@osu.edu*) o con Magdiel Lopez Soriano de la Universidad de Missouri al (*573-445-8375 o mlopezsoriano@missouri.edu*). Si tiene preguntas sobre sus derechos como participante de la investigación, comuníquese con la oficina del Consejo de Revisión Institucional de la Universidad de Missouri (IRB) al *573-882-3181 o muresearchirb@missouri.edu*. El IRB es un grupo de personas que revisan estudios de investigación para garantizar que los derechos y el bienestar de los participantes estén protegidos.

Puede pedirle al investigador que le proporcione una copia de este consentimiento para sus registros, o puede guardar una copia de este consentimiento si ya se la ha proporcionado. Agradecemos su consideración de participar en este estudio.

P1. ¿Consiente en tomar la encuesta?

☐ SI → *siga por favor* ☐ NO → *por favor devuelve la encuesta*

P2. ¿En qué estado usted trabaja actualmente?

☐ Iowa ☐ Illinois ☐ Minnesota ☐ Missouri ☐ Ohio

☐ Otro (especifique): ____

2a: ¿para qué compañía trabaja actualmente?: __________________________________

P3. ¿Cuánto tiempo llevas participando como TN en Estados Unidos? *(Por ejemplo: 5 años y 3 meses)*

Años: _______ Meses: _______

P4. ¿Trabajó en otras industrias antes de venir a trabajar con cerdos tanto dentro como fuera de los EE. UU.?

☐ SI → *siga por favor* ☐ NO → *salta a la P5*

4a. **En caso de responder si, en cuales de las siguientes industrias trabajó:** *(marque todo lo que aplique)*

☐ Equinos
☐ Ganado de Leche
☐ Ganado de Carne
☐ Avícola
☐ Ovejas o Cabras
☐ Cultivos
☐ Invernaderos
☐ Plantas empacadoras o de sacrificio
☐ Otro (especifique): ____

P5. ¿Cuáles son las 3 cosas que más le gustan de trabajar en una granja de cerdos?

1) ____

2) ____

3) ____

P6. ¿Cuáles son las razones por las que viene a trabajar a la granja todos los días?

1) ____

2) ____

3) ____

P7. ¿Cuál es el tamaño de la granja en la que trabaja actualmente (cuántos cerdos)?

☐ 1000 o menos ☐ 1001 – 2500 ☐ 2501 – 5000 ☐ 5001 o mas

P8. ¿Cuál es su puesto en la granja porcina?

☐ Empleado por hora
☐ Gerente de producción en entrenamiento
☐ Jefe de departamento o jefe de equipo
☐ Gerente de granja (una granja)
☐ Gerente de producción (varias granjas)
☐ Otro (especifique): ______________________________

P9. ¿En qué zona de la granja pasa la mayor parte de su tiempo? *Marque todo lo que aplique:*

☐ Maternidad
☐ Inseminación
☐ Gestación
☐ GDU (primerizas)
☐ Criadero (nursery)
☐ Finalización
☐ Otro (especifique): ______________________________

P10. ¿Está actualmente satisfecho o insatisfecho en su granja?

- ☐ Muy satisfecho
- ☐ De alguna manera satisfecho
- ☐ Ni satisfecho ni insatisfecho
- ☐ De alguna manera insatisfecho
- ☐ Muy insatisfecho

P11. Por favor explique qué lo hace satisfecho o insatisfecho.

____

____

____

P12. ¿Ha pensado en cambiar de su empleador actual?

☐ SI → *por favor explique por qué a continuación* ☐ NO → *salta a la P13*

P12a. ¿Por qué pensaba en cambiar de su empleador?

____

____

____

P13. ¿Hasta qué punto siente que su trabajo actual es estresante?

- ☐ Nada estresante
- ☐ Mayormente no estresante
- ☐ Ni muy estresante ni poco estresante
- ☐ Un poco estresante
- ☐ Muy estresante

P13a. Si siente estrés, por favor explique por qué:

____

____

____

Extension
University of Missouri

P14. **¿Qué beneficios <u>recibe actualmente</u> como empleado?** *(Por favor marque todo lo que aplique)*

- ☐ Seguro médico o de salud
- ☐ Plan de retiro
- ☐ Bono de producción
- ☐ Asistencia a entrenamientos especiales o conferencias
- ☐ Vacaciones pagadas (PTO)
- ☐ Bono por recomendación
- ☐ Otro (especifique): ____

P14. ¿Qué tan importante son los siguientes beneficios que <u>recibe actualmente</u>? *Marque en el cuadro que mejor aplique a usted. Si no recibe un tipo de beneficio – marque el circulo a la derecha.*

	**No es tan importante**	**Un poco importante**	**Importante**	**Muy Importante**	**No Recibo Este Beneficio**
Seguro médico o de salud	☐	☐	☐	☐	○
Plan de retiro	☐	☐	☐	☐	○
Bono de producción	☐	☐	☐	☐	○
Asistencia a entrenamientos especiales o conferencias	☐	☐	☐	☐	○
Vacaciones pagadas (PTO)	☐	☐	☐	☐	○
Bono por recomendación	☐	☐	☐	☐	○
Otro (especifique): ______________	☐	☐	☐	☐	○

P15. Si su empleador agregara <u>nuevos beneficios</u>, ¿cuáles de los siguientes beneficios le gustaría obtener más? *(marque todo lo que aplique)*

- ☐ Seguro médico o de salud
- ☐ Plan de retiro
- ☐ Bono de producción
- ☐ Asistencia a entrenamientos especiales o conferencias
- ☐ Vacaciones pagadas (PTO)
- ☐ Bono por recomendación
- ☐ Otro (especifique): ____

P15a. ¿Qué tan importante son cada uno de los siguientes nuevos beneficios posibles que le gustaría recibir? *Marque en el cuadro que mejor aplique a usted. Si ya recibe un tipo de beneficio – marque el círculo a la derecha.*

	**No es tan Importante**	**Un poco Importante**	**Importante**	**Muy Importante**	**Ya Recibo Este Beneficio**
Seguro médico o de salud	☐	☐	☐	☐	○
Plan de retiro	☐	☐	☐	☐	○
Bono de producción	☐	☐	☐	☐	○
Asistencia a entrenamientos especiales o conferencias	☐	☐	☐	☐	○
Vacaciones pagadas (PTO)	☐	☐	☐	☐	○
Bono por recomendación	☐	☐	☐	☐	○
Otro (especifique): ______________ ______________	☐	☐	☐	☐	○

P16. ¿Qué tan importante son cada una de las siguientes razones en su decisión trabajar en los EE. UU con una visa TN?

	No es tan importante	Un poco importante	Importante	Muy Importante
Para obtener un mejor salario	☐	☐	☐	☐
Para apoyar económicamente a mi familia en México	☐	☐	☐	☐
Por falta de oportunidades en México.	☐	☐	☐	☐
Obtener beneficios como asistencia de salud, retiro, etc.	☐	☐	☐	☐
Para obtener entrenamiento y desarrollar mis habilidades profesionales	☐	☐	☐	☐
Para aprender o mejorar mi nivel de inglés	☐	☐	☐	☐
Otro (especifique): ________________	☐	☐	☐	☐

P17. ¿Qué tan importante son cada uno de las siguientes metas y aspiraciones para usted en su carrera profesional?

	No es tan importante	Un poco importante	Importante	Muy Importante
Un incremento en salario	☐	☐	☐	☐
Aplicar a un ascenso laboral	☐	☐	☐	☐
Ganar experiencia y luego regresar a México	☐	☐	☐	☐
Solicitar un trabajo en otra empresa	☐	☐	☐	☐
Seguir estudiando y obtener otro diploma	☐	☐	☐	☐
Otro (especifique): ________________	☐	☐	☐	☐

P18. ¿Qué tan importante son cada una de las siguientes metas personales para usted?

	No es tan importante	Un poco importante	Importante	Muy Importante
Traer a mi familia a los EE. UU	☐	☐	☐	☐
Obtener un estatus migratorio permanente	☐	☐	☐	☐
Quedarme en esta finca hasta la jubilación	☐	☐	☐	☐
Ahorrar Dinero	☐	☐	☐	☐
Otro (especifique): ____________________	☐	☐	☐	☐

P19. ¿Con qué frecuencia recibe (o participa en) capacitación en su granja actual? *No incluya reuniones de producción*

☐ Semanalmente ☐ Mensualmente ☐ Anualmente ☐ Nunca

☐ Otro (especifique): ____

P20. ¿Cuántas oportunidades de entrenamiento recibe actualmente de su empleador? *No incluya reuniones de producción*

____

P21. ¿Qué tan importante son cada uno de los siguientes tipos de oportunidades potenciales de entrenamiento para usted?

	No es tan importante	Un poco importante	Importante	Muy importante
Desarrollar habilidades para trabajar con cerdos	☐	☐	☐	☐
Manejo de personal	☐	☐	☐	☐
Resolución de conflictos	☐	☐	☐	☐
Estrategias para apoyar el cuidado personal y el bienestar	☐	☐	☐	☐
Manejo de finanzas personales	☐	☐	☐	☐
Otro (especifique): ____________________	☐	☐	☐	☐

P22. ¿Está usted al tanto de las oportunidades de promoción en el trabajo?

☐ **Si** ➔ *siga* ☐ **No** ➔ *salta a P24* ☐ **No estoy seguro** ➔ *siga*

P23. ¿Cuáles de los siguientes puestos/oportunidades para ascenso son disponibles en su granja porcina actual? *Marque todo lo que aplica*

☐ Gerente de producción en entrenamiento
☐ Jefe de departamento o jefe de equipo
☐ Gerente de granja (una granja)
☐ Gerente de producción (varias granjas)
☐ Auditor (bienestar animal, seguridad ocupacional, ambiental)
☐ Recursos humanos (Gerente, Generalista, Administrativo)
☐ Entrenador técnico de producción
☐ Otro (especifique): ____

P24. Si se presentara la oportunidad de postular a un ascenso, ¿usted se postularía?

☐ **Si** ➔ *salta a la P25* ☐ **No** ➔ *siga* ☐ **No estoy seguro**

P24a En caso de responder no, elija la razón principal de las siguientes opciones: *(marca uno)*:

☐ No me siento seguro de la capacitación recibida con mi empleador actual para solicitar un ascenso.
☐ No tengo suficiente experiencia para aplicar a un mejor puesto
☐ No tengo suficiente educación para aplicar a un mejor puesto
☐ No estoy interesado en aplicar a una promoción
☐ Mi aplicación no será tomada en consideración
☐ Otro (especifique): ____

P25. ¿Qué tan difíciles son cada uno de los siguientes aspectos de establecerse o adaptarse a los EE. UU?

	No fue muy difícil	Un poco difícil	Difícil	Muy Difícil
Barreras culturales y de lenguaje	☐	☐	☐	☐
Comprar una forma de transporte confiable	☐	☐	☐	☐
Encontrar vivienda asequible	☐	☐	☐	☐
Obtener una licencia de conducir en EE. UU.	☐	☐	☐	☐
Ahorrar dinero	☐	☐	☐	☐
Otro (especifique): ____________________	☐	☐	☐	☐

P26. ¿Cuál es el nivel más alto de educación que ha completado o el título más alto que ha recibido?

☐ Licenciatura
☐ Certificación (más allá de la licenciatura)
☐ Grado de especialización
☐ Maestría
☐ Doctorado
☐ Otro (especifique): ____

P27. ¿En qué área de estudio se graduó?

☐ Agronomía
☐ Producción animal
☐ Medicina veterinaria
☐ Biología/Ecología
☐ Otro (especifique): ____

P28. ¿En qué idioma se siente más cómodo hablando?

☐ Inglés
☐ Español
☐ Dialecto nativo
☐ Otro (especifique): ______________________________

P29. ¿Qué tan cómodo se siente hablando inglés?

☐ Básico (solo conoce algunas palabras y frases)
☐ Intermedio (puede hablar con relativa fluidez con otros)
☐ Avanzado (puede tener conversaciones completas y sentirse seguro al hablar inglés)

P30. Cuando la gente le habla inglés, ¿cuánto entiendes?

☐ Muy poco ☐ Un poco ☐ Algo ☐ La mayoría ☐ Todo

P31. Cuando habla inglés, ¿cuánto crees que entienden los demás?

☐ Muy poco ☐ Un poco ☐ Algo ☐ La mayoría ☐ Todo

P32. ¿Con que frecuencia se le proporciona capacitación, procedimientos operativos estándar (SOP) o literatura escrita en español?

☐ Nunca ☐ Casi nunca ☐ A veces ☐ La mayor parte del tiempo ☐ Todo el tiempo

P33. ¿Cuál es tu género o sexo?

☐ M ☐ F ☐ Prefiere no decir
☐ Prefiere autodefinirse: ______________________________

P34. ¿Cuántos años tiene?

☐ 18 - 25 ☐ 26 - 34 ☐ 35 - 44 ☐ 45 – 54 ☐ 55 – 64 ☐ 65 o más
☐ Prefiere no decir

P35. ¿Cómo describirías el área en la que pasaste la mayor parte de tu vida?

☐ Ciudad (100.000 o más personas)
☐ Suburbano/Ciudad grande (20.000-100.000)
☐ Pueblo pequeño (5.000 - 20.000)
☐ Zona rural (5.000 o menos)

Extension
University of Missouri

P36. ¿Hay algo más que le gustaría compartir? *Por favor escriba comentarios adicionales aquí.*
